# Antibody-Mediated Inhibition of TNFR1 Attenuates Disease in a Mouse Model of Multiple Sclerosis

**DOI:** 10.1371/journal.pone.0090117

**Published:** 2014-02-28

**Authors:** Sarah K. Williams, Olaf Maier, Roman Fischer, Richard Fairless, Sonja Hochmeister, Aleksandar Stojic, Lara Pick, Doreen Haar, Sylvia Musiol, Maria K. Storch, Klaus Pfizenmaier, Ricarda Diem

**Affiliations:** 1 Department of Neuro-oncology, University Clinic Heidelberg, Heidelberg, Germany; 2 Institute of Cell Biology and Immunology, University of Stuttgart, Stuttgart, Germany; 3 Department of Neurology, Medical University of Graz, Graz, Austria; 4 Department of Neurology, University of the Saarland, Homburg/Saar, Germany; Friedrich-Alexander University Erlangen, Germany

## Abstract

Tumour necrosis factor (TNF) is a proinflammatory cytokine that is known to regulate inflammation in a number of autoimmune diseases, including multiple sclerosis (MS). Although targeting of TNF in models of MS has been successful, the pathological role of TNF in MS remains unclear due to clinical trials where the non-selective inhibition of TNF resulted in exacerbated disease. Subsequent experiments have indicated that this may have resulted from the divergent effects of the two TNF receptors, TNFR1 and TNFR2. Here we show that the selective targeting of TNFR1 with an antagonistic antibody ameliorates symptoms of the most common animal model of MS, experimental autoimmune encephalomyelitis (EAE), when given following both a prophylactic and therapeutic treatment regime. Our results demonstrate that antagonistic TNFR1-specific antibodies may represent a therapeutic approach for the treatment of MS in the future.

## Introduction

Multiple sclerosis (MS) is a chronic inflammatory disease of the central nervous system (CNS) and the most frequent cause of neurological disability in young adults. Until recently, it has been primarily thought of as an autoimmune inflammatory demyelinating disease, however in the last decade it has become clear that neurodegeneration is the underlying pathological cause of permanent disability [1]–[3].

TNF is a master proinflammatory cytokine that exists as both membrane bound and soluble isoforms and plays a dominant role in the initiation and perpetuation of chronic inflammation [4]. It has been implicated in the pathology of many autoimmune diseases and anti-TNF therapies are successfully used to treat autoimmune diseases such as rheumatoid arthritis, Crohn's disease and psoriasis [5].

The role played by TNF in the pathology of MS, however, appears to be complex. In MS patients, both serum and CSF levels of TNF are elevated [6] and appear to correlate with the severity of symptoms [7]. Additionally, both TNF and its two receptors, TNFR1 and TNFR2, are all upregulated in MS lesions [8], [9]. The deleterious effect of TNF in MS has been further emphasized by animal studies showing that TNF inhibition reduced the severity of experimental autoimmune encephalomyelitis (EAE) symptoms [10], [11].

Given these findings, the transfer of anti-TNF therapies to the clinic led to unexpected results. Trials of non-selective TNF inhibitors had to be halted due to a worsening of neurological symptoms compared to patients treated with placebo [12], [13]. Furthermore, it was subsequently revealed that a number of rheumatoid arthritis patients treated with anti-TNF therapy developed neurological symptoms, including demyelinating lesions [14], [15].

Since then, it has become clear that TNF mediates specific and often opposing effects via TNFR1 and TNFR2. TNFR1, predominantly activated by soluble TNF [16], exerts proinflammatory effects [17], [18], whereas TNFR2, preferentially activated by membrane bound TNF [16] promotes both neuroprotection and remyelination [19], [20].

Therefore, whilst TNF remains a potential therapeutic target for the treatment of MS and other neuroinflammatory disorders, its targeting should be strictly selective. As such, the validity of specifically targeting TNFR1 as a therapeutic approach in animal models of MS has recently been verified. Both a TNFR1-selective antagonistic mutant TNF (R1antTNF) [21], [22] and a soluble dominant-negative TNF (XPro1595), were shown to exert beneficial effects in EAE [23], [24]. Furthermore, inhibition of the pre-ligand assembly domain of TNFR1 was shown to ameliorate spinal cord symptoms and downregulate the Th17 response in mice [25].

Since antibodies are known to be superior to cytokines with respect to pharmacokinetic and pharmacodynamic properties, here we have determined the effect of a mouse TNFR1-specific antagonistic antibody on the course of MOG^35–55^-induced EAE in C57BL/6 mice. We show that a single injection of the antibody at the time of immunization is sufficient to delay and ameliorate the disease, which is accompanied by reduced demyelination of the spinal cord. Moreover, in a therapeutic setting, i.e. application after disease onset, we show that anti-TNFR1 treatment also significantly reduces EAE symptoms.

## Materials and Methods

### Ethics statement

All experiments that involved animal use were performed in strict compliance with the relevant laws and institutional guidelines. The protocols and procedures have been approved by the Landesamt für Gesundheit und Verbraucherschutz, Saar-Pfalz Kreis, Germany (Az:c1-2.4.2.2/09/2011) and Regierungspräsidium Karlsruhe, Germany (Az.35-9185.81/G-35/12).

### Animals

Female C57BL/6 mice of 6 to 8 weeks of age were used in all experiments. TNFR1^-/-^ [26] and TNFR2^-/-^ [27] mice were from Horst Bluethmann (Hoffmann-La Roche, Basel, Switzerland) and were backcrossed to a C57BL/6 background a minimum of 20 generations. Homozygosity of these mice was verified by genotyping as described previously [20]. Animals were kept under environmentally-controlled conditions in the absence of pathogens.

### Evaluation of acute TNF toxicity *in vivo*

Recombinant mouse TNF (Immunotools, Friesoythe, Germany) was injected intravenously (i.v.) into female C57BL/6 wild type mice (1 mg/kg in 100 µl PBS). To evaluate the capability of anti-TNFR1 treatment to block acute TNF toxicity we used the antibody HM1097 (Hycult Biotech, Uden the Netherlands), which has been described previously as a specific antagonist for mouse TNFR1 (55R-170), [28]. HM1097 was injected intraperitoneally (i.p.) two hours prior to the TNF injection. Control animals received PBS. The body weight and temperature as well as behavioral changes of the animals were monitored to evaluate the toxic effect of TNF.

### Induction and evaluation of EAE

Female mice, 6 to 8 weeks of age, were immunized subcutaneously in the flanks with 300 µg MOG^35–55^ in phosphate buffered saline (PBS) emulsified in an equal volume of complete Freund's adjuvant (CFA, Sigma-Aldrich, St. Louis, MO) supplemented with 1 mg *Mycobacterium tuberculosis* H37RA (Difco, Detroit, Michigan). Immediately afterwards, and again 48 hours later, mice received i.p. injections of 300 ng pertussis toxin (List Biological Labs, Campbell, CA). Animals were weighed and scored on a daily basis. Disease severity was assessed using a scale ranging from 0 to 5: 0, no clinical disease; 0.5, distal paresis of the tail; 1.0, complete paralysis of the tail; 1.5, paresis of tail and slightly impaired righting; 2.0, gait ataxia and severely reduced righting; 2.5, bilateral severe hind limb paresis; 3.0, complete bilateral hind limb paralysis; 3.5, complete bilateral hind limb paralysis and weakness of forelimbs; 4, paralysis of hind limbs and paresis of fore limbs; 4.5, paralysis of hind limbs and paralysis of fore limbs; 5, moribund state or death.

### Treatment of animals

Mice were injected i.p. with either anti-mouse TNFR1 (HM1097, Hycult Biotech, Uden, The Netherlands) or an isotype control antibody (Armenian Hamster IgG negative control, AbD Serotec, Düsseldorf, Germany) diluted in saline. Injections were given following two regimes; a prophylactic regime immediately following disease induction or according to a therapeutic regime, on the first day of clinical symptoms (EAE day 1) and again three days later (EAE day 4). A minimum of 4 mice were included per treatment group, and each treatment was repeated at least in duplicate.

### Histopathology

Mice received an overdose of ketamine/xylazine and were transcardially perfused with 4% paraformaldehyde in PBS. Spinal cords were removed and processed for paraffin-embedding. Histopathological evaluation was performed on 0.5 µm paraffin-embedded transverse sections of the spinal cord. Luxol fast blue (LFB) staining was performed in order to assess demyelination and immunohistochemistry was performed in order to assess inflammatory infiltration. For immunohistochemistry, following antigen retrieval in citrate buffer and blocking, antibodies against Mac-3 (1∶200, BD Pharmingen, San Diego, CA) to detect activated microglia/macrophages, CD3 (1∶150, Dako, Glostrup, Denmark) to detect T cells, β-amyloid precursor protein (β-APP; 1∶000, Millipore, Billerica, MA) to detect injured axons and NeuN (1∶400, Milipore) to detect surviving neurons in the spinal cord grey matter, were diluted in the appropriate serum and incubated overnight at 4°C. For β-APP, antigen retrieval was performed by steaming in a pressure cooker for 1 hour followed by 1 hour cooling. After incubation in biotinylated secondary antibodies, an avidin-biotin amplification was performed (ABC Elite kit, Vector, Burlinghame, CA).

### Analysis of spinal cord lesions

Spinal cord histopathology was analysed as previously described [29]. The degree of demyelination was evaluated semi-quantitatively using the following scoring system: traces of perivascular or subpial demyelination (0.5); marked perivascular or subpial demyelination (1); confluent perivascular or subpial demyelination (2); demyelination of half spinal cord cross-section (3); transverse myelitis (4). The quantitative assessment of Mac-3 and CD3-positive cells, β-APP-positive axons and NeuN-positive neurons was achieved by determining the number of positive cells, axonal profiles or neuronal somata in an average of 15 complete spinal cord cross sections per animal. These values were then converted to mm^2^.

### *In vitro* characterization of anti-TNFR1

L929 fibroblasts were cultivated in 96 well plates (20,000 cells/well) in RPMI 1640 medium supplemented with 5% FCS and 100 U/ml penicillin and 100 µg/ml streptomycin (Life Technologies, Darmstadt, Germany) overnight. Then the cells were incubated with 1 µM of the RNA transcription inhibitor actinomycin D for 30 min in presence or absence of HM1097 before addition of murine TNF. After 20 h the cells were washed with PBS and incubated with crystal violet (0.5% crystal violet in 20% methanol) for 20 minutes to stain viable cells. The dye was washed away under rinsing water and the cells were air-dried. Crystal violet was resolved with methanol and the optical density at 550 nm was determined. Each sample was analyzed in triplicate and data were analyzed using the software Microsoft Excel and GraphPad Prism 4.

### Statistical analyses of EAE

All data are presented +/− SEM. The cumulative score was calculated as the sum of the daily scores. Statistical comparisons were made using SigmaPlot 12 (Systat Software GmbH, Ekrath, Germany). Data was assessed for normality using the Shapiro-Wilk Test, and subsequently analysed using Mann-Whitney (for ordinal EAE scores, and histopathological analyses of TNFR1^-/-^, TNFR2^-/-^ and WT mice), or the Student's t-test. A p value of <0.05 was considered statistically significant.

## Results

### EAE severity depends upon TNFR expression

**Figure 1:**
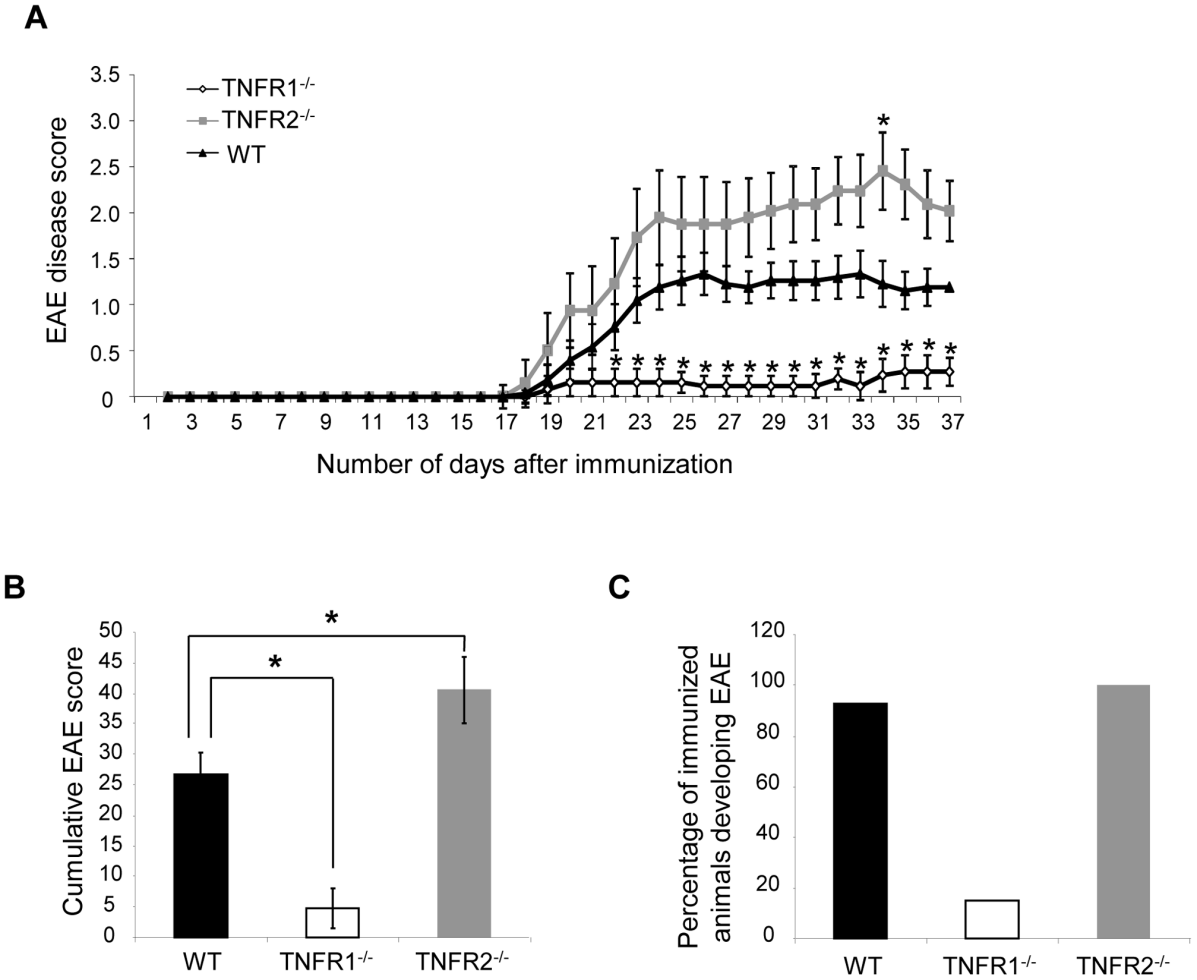
EAE severity depends upon TNFR expression. EAE was induced with MOG^35–55^ in TNFR1^-/-^, TNFR2^-/-^ and WT mice and the resulting disease was assessed daily using a score from 0 to 5, until 21 days after the onset of neurological symptoms. TNFR1^-/-^ mice had a much less severe disease course when compared to WT mice, whereas TNFR2^-/-^ mice showed more severe symptoms than WT mice (**A**). These observations were reflected in the significantly reduced cumulative EAE score in TNFR1^-/-^ mice, and the significantly increased cumulative score in TNFR2^-/-^ mice (**B**). Furthermore, TNFR1^-/-^ mice were more resistant to disease induction than both TNFR2^-/-^ and WT mice (**C**). * P<0.05, TNFR1^-/-^ n = 13; TNFR2^-/-^ n = 8; WT n = 14.

**Figure 2:**
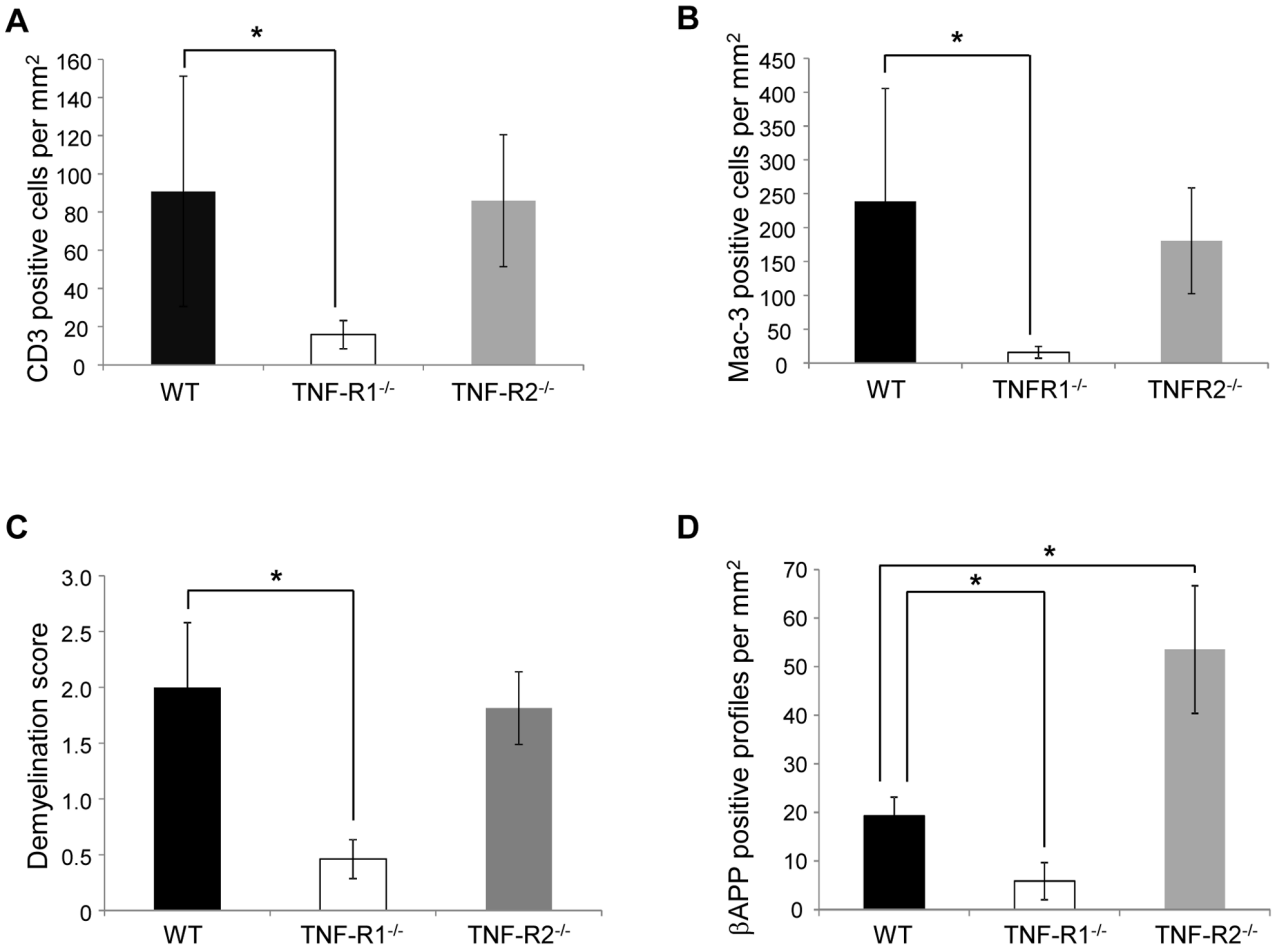
Histopathological analysis confirmed that EAE was less severe in TNFR1^-/-^ mice in comparison to both WT and TNFR2^-/-^ mice. Spinal cords were analysed at day 21 of EAE for signs of inflammatory infiltration, demyelination and axonal degeneration. TNFR1^-/-^ mice had significantly less infiltration of both CD3-positive T cells (**A**) and Mac-3-positive activated microglia/macrophages (**B**) when compared to WT and TNFR2^-/-^ mice. Furthermore, TNFR1^-/-^ mice also had significantly less demyelination, as assessed by LFB staining (**C**) and axonal damage, as assessed by accumulation of β-APP-positive axonal profiles (**D**), when compared to both WT and TNFR2^-/-^ mice. Conversely, TNFR2^-/-^ had significantly increased numbers of β-APP positive damaged axons compared to WT mice.

In an initial experiment, we sought to determine the role of the two TNF receptors in the development of EAE induced by MOG^35–55^. Mice deficient for either TNFR1 or TNFR2, as well as wild type (WT) mice were immunized with MOG^35–55^ and scored daily until 21 days after the onset of spinal cord symptoms, according to a scale from 0 to 5. As has been previously described [30]–[32], we found that TNFR1^-/-^ mice were almost completely protected from EAE as demonstrated by a strongly reduced EAE incidence and EAE disease score compared to WT mice (Fig. 1). Accordingly, in the spinal cord of TNFR1^-/-^ mice, activation of macrophages as well as infiltration of CD3-positive T-cells was highly reduced compared to wild type mice (Fig. 2A, B). This was accompanied by strongly reduced demyelination and neurodegeneration as determined by LFB-staining and immunohistochemistry for β-APP-positive axons, respectively (Fig. 2C, D). In contrast, EAE symptoms were exacerbated in TNFR2^-/-^ mice (Fig. 1). Although activation and infiltration of immune cells was not altered compared to wild type mice, lack of TNFR2 resulted in increased neurodegeneration in the spinal cord (Fig. 2D) as assessed by accumulation of the fast axonal transport protein β-APP. These results confirm previous findings that TNFR1 is essential for EAE development whereas TNFR2 appears to have a neuroprotective role in this disease model [30], [31].

Due to the more striking effects of TNFR1 ablation on EAE-related parameters than TNFR2, this prompted us to determine whether inhibiting TNFR1 with an antibody specific for mouse TNFR1 could ameliorate EAE symptoms in C57BL/6 WT mice. For this purpose we used an antibody from Armenian Hamster which has been previously characterized as antagonistic towards mouse TNFR1 [28].

### Selective inhibition of TNFR1 prevents acute TNF toxicity *in vitro* and *in vivo*

**Figure 3:**
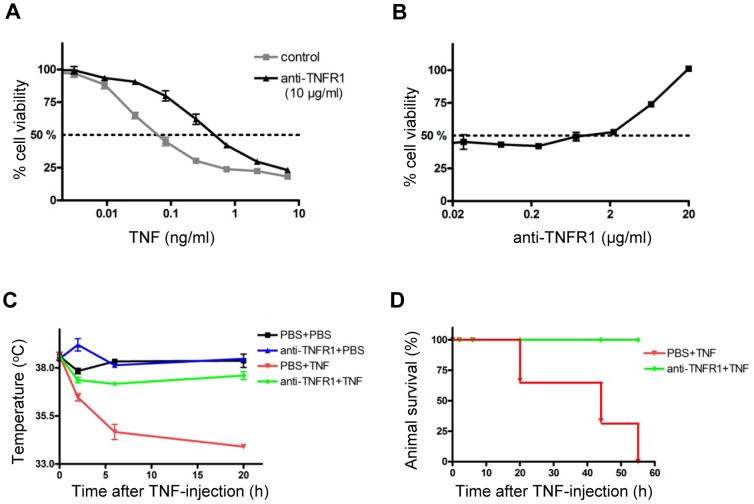
Anti-TNFR1 inhibited acute TNF toxicity *in vitro* and *in vivo*. L929 cells were incubated with recombinant mouse TNF in the presence or absence of 10 µg/ml anti-TNFR1 (**A**) or with anti-TNFR1 in the presence of 0.1 ng/ml mouse TNF (**B**) and cell survival was determined by crystal violet staining. **C, D**: Female C57BL/6 wild type mice were pretreated with PBS or anti-TNFR1 (10 mg/kg, i.p.). After 2 h, PBS or murine TNF were injected i.v. (1 mg/kg) and body temperature (**C**) as well as survival of animals (**D**) were determined.

We first verified the neutralizing activity of the anti-mouse TNFR1 antibody on L929 cells. In presence of actinomycin D, TNF has a strong toxic effect on L929 cells with an EC_50_ of below 0.1 ng/ml (Fig. 3A). Addition of 10 µg/ml HM1097 resulted in an almost 10-fold increase of the EC_50_ demonstrating the antagonistic effect of the antibody on TNF-induced toxicity *in vitro* (Fig. 3A). To determine the concentration at which the antibody protects against TNF toxicity *in vitro*, we next titrated the antibody and analyzed its effect when L929 cells were treated with 0.25 ng/ml TNF. In this setting, HM1097 started to neutralize TNF at a concentration of 2 µg/ml (Fig. 3B),

After verifying that HM1097 can antagonize TNFR1 activation *in vitro* we next analyzed the effect of this antibody on acute TNF toxicity *in vivo*. To this end we injected a lethal dose of mouse TNF into female C57BL/6 mice (1 mg/kg, i.v.). All mice developed severe pathology and within several hours showed pathological symptoms such as piloerection, tremor and ataxia. Moreover, the body temperature dropped below 35°C (Fig. 3C) resulting ultimately in the death of all mice (Fig. 3D). Importantly, pretreatment of the mice with HM1097 (10 mg/kg, i.p.) prevented TNF toxicity. Anti-TNFR1 treated mice showed no overt pathological symptoms with only a mild transient reduction of body temperature after TNF injection and all mice survived the TNF-treatment (Fig. 3C, D).

### Treatment with anti-mouse TNFR1 following a prophylactic treatment regime delays disease onset and reduces EAE severity

Given the dramatic effect of TNFR1 ablation on EAE disease progression and the antagonistic effect of the mouse TNFR1 specific antibody, we decided to determine the therapeutic effect of this antibody in the MOG^35–55^–induced EAE in C57BL/6 WT mice using different treatment paradigms. In a first approach, mice were treated with 100 µg (equivalent to 5 mg/kg) of either anti-mouse TNFR1 or an isotype control antibody immediately following induction of EAE with MOG^35–55^. The resulting EAE was monitored and scored daily until 21 days after the onset of clinical symptoms, according to a scale from 0 to 5.

**Figure 4:**
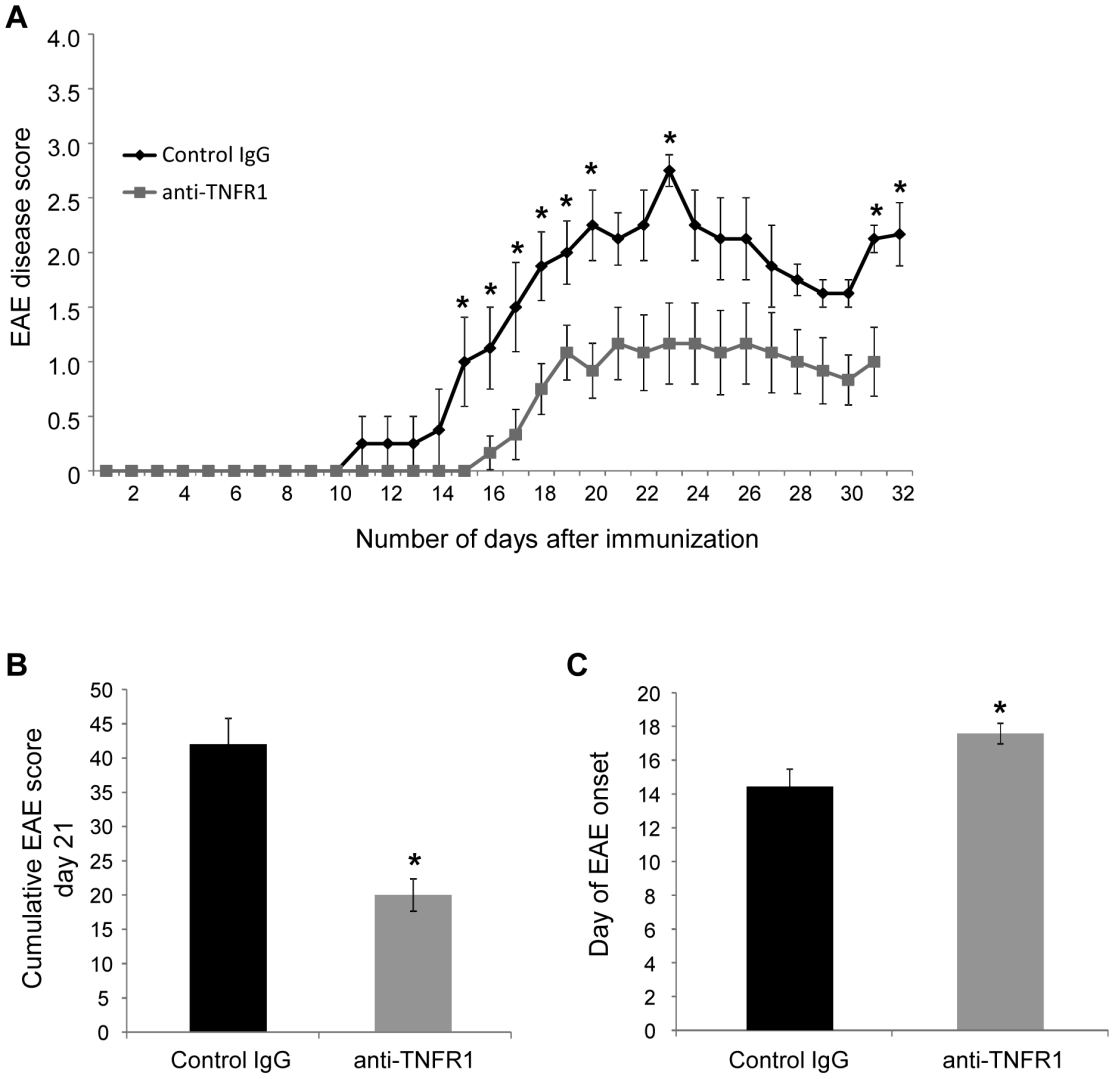
Administration of anti-mouse TNFR1 on the day of immunization ameliorated EAE. Anti-mouse TNFR1 was injected intra-peritoneally in C57BL/6 mice, on the day of disease induction, at a dosage of 100 µg (equivalent to 5 mg/kg). Mice were subsequently monitored on a daily basis until 21 days after the onset of clinical symptoms (EAE day 21). Antibody treatment resulted in a reduced EAE severity compared to mice receiving control IgG (**A, B**). Furthermore, mice injected with anti-TNFR1 also showed a significant delay in the onset of spinal cord symptoms in comparison to mice receiving control IgG (**C**). (A) Results from one representative experiment out of four shown (control IgG n = 4; anti TNFR1 n = 6), (B, C) results from four combined experiments (control IgG n = 16, anti-TNFR1 n = 19). * P<0.05, **P<0.01.

Mice receiving this single injection of anti-mouse TNFR1 on the day of immunization had a less severe EAE compared to wild type mice (Fig. 4A) resulting in a significant reduction in the overall cumulative score (Fig. 4B). Moreover, a significant delay of disease onset of approximately 3 days was observed (Fig. 4C). A summary of the characteristics of the resultant EAE is given in Table 1. Some variation in disease severity was apparent between EAE scores presented in Figure 1, possible resulting from alterations in animal holding facilities and immunisation reagents, which are known to influence disease course [33]. However, all comparative experiments were performed simultaneously under identical conditions.

**Table 1:** Characteristics of EAE following anti-TNFR1 treatment from the day of immunization.

	Control IgG	Anti-TNFR1
**Incidence of EAE**	100%	100%
**Day of onset**	14.56±0.96	17.53±0.63
**Maximum mean disease score**	2.2±0.26	1.32±0.84
**Cumulative disease score to day 21**	42±3.79	19.9±2.35

**Figure 5:**
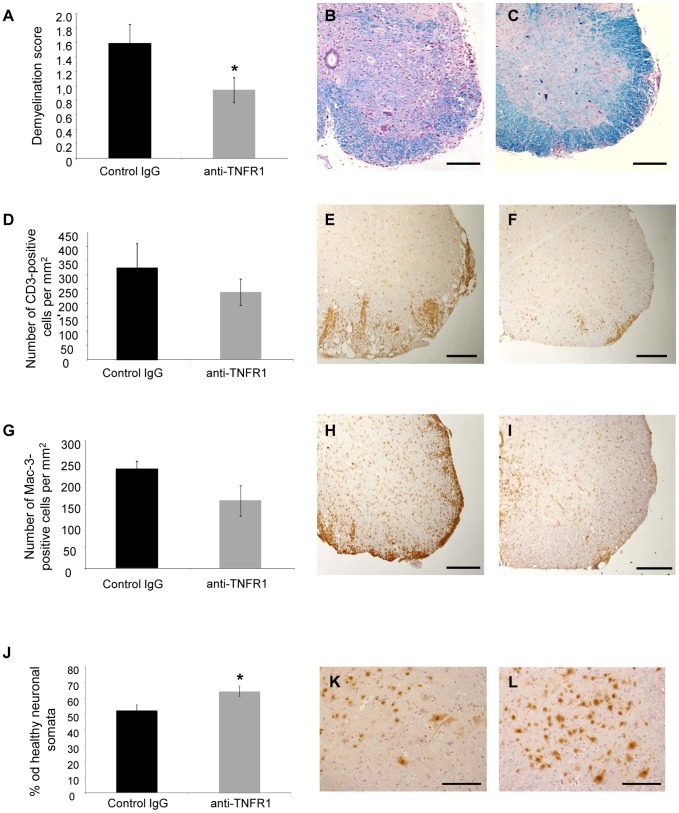
Anti-TNFR1 treatment on the day of immunization resulted in a significant reduction in demyelination and neuronal loss and a mild reduction in inflammatory infiltration. Spinal cord histopathology was performed at day 21 of EAE, following treatment with anti-mouse TNFR1 on the day of immunization. Representative images are shown from control IgG treated mice, with an EAE score of 2.0 (**B, E, H**, and **K**) and from anti-TNFR1-treated animals, with an EAE score of 1.0 (**C, F, I** and **L**). The level of spinal cord demyelination was assessed using sections stained with LFB (**A–C**). Mice treated prophylactically with anti-TNFR1 had significantly reduced levels of demyelination compared to control-treated mice (**A–C**). Immunohistochemistry with an anti-CD3 antibody was used to detect T cells and showed a decrease, although not significant, in the number of T cells within the spinal cord of anti-TNFR1 treated mice, in comparison to control animals (**D–F**). Immunohistochemistry with an antibody to Mac-3 was used to detect activated microglia and macrophages and demonstrated a decrease in the number of positive cells in anti-TNFR1 treated mice, although again this was not significant (**G–I**). **J-L**: Immunohistochemistry with an anti-NeuN antibody was used to detect neuronal cell bodies, which were quantified within the spinal cord grey matter. Anti-TNFR1 treated mice had significantly elevated numbers of surviving neuronal cell bodies. Scale bars in **B, C, E, F, H, I, K, L**: 200 µm.

Spinal cords were removed at day 21 of EAE and analysed histopathologically. Corresponding to the reduced disease severity, mice treated with anti-TNFR1 on the day of immunization showed less severe spinal cord demyelination than control animals (Fig. 5A–C). Immunohistochemistry with an antibody against CD3, however, showed only a mild reduction in CD3-positive T cell infiltration in anti-TNFR1-treated mice compared to control IgG-treated mice, which was not significant (Fig. 5D–F). This result was reflected in the observation that anti-TNFR1-treated mice had only a trend towards reduced activated microglia/macrophage infiltration, as indicated by immunohistochemistry using an antibody to Mac-3 (Fig. 5G–I). However, staining for neuronal cell bodies in the grey matter of the spinal cord using an antibody against NeuN revealed significant preservation of neurons in the anti-TNFR1-treated group (Fig. 5J–L).

### Treatment with anti-mouse TNFR1 following a therapeutic treatment regime reduces EAE severity

After this positive effect on EAE development in a prophylactic setting, we investigated whether anti-TNFR1 treatment could also reduce EAE severity according to a therapeutic regime. For this, mice were immunized with MOG^35–55^ as previously described, and treated with different dosages of either anti-mouse TNFR1 or an isotype control antibody on the first day of neurological symptoms. The subsequent disease was monitored and scored daily for 14 days, according to a scale from 0 to 5.

**Figure 6:**
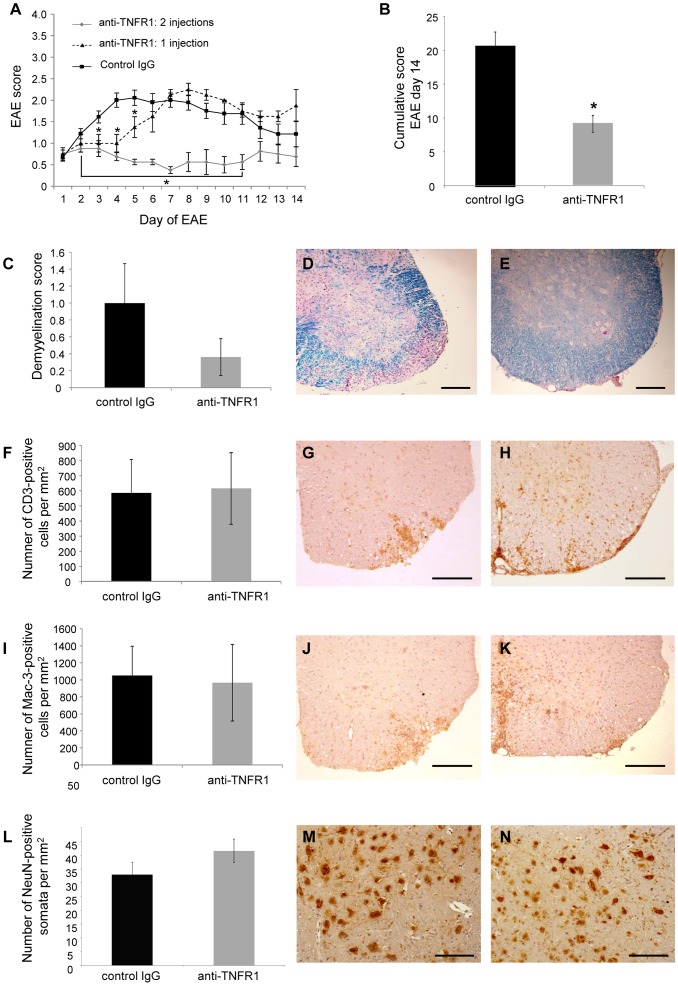
Anti-TNFR1 treatment reduced EAE severity in a therapeutic setting. EAE was induced in C57BL/6 mice with MOG^35–55^ and mice were assessed for neurological symptoms on a daily basis. 400 µg (equivalent to 20 mg/kg) anti-mouse TNFR1 was injected i.p. either once on the day of EAE onset, or twice; firstly on the day of EAE onset and again on day 4 of the disease. Control mice received two injections of control IgG on days 1 and 4 of EAE. Mice receiving two injections of anti-TNFR1 had significantly reduced neurological symptoms than mice receiving control IgG (**A**, **B**). Spinal cords were examined histopathologically at day 14 of EAE by LFB staining (**C**–**E**). Control IgG-treated mice (**D**, image from animal with an EAE score of 2.5) had more demyelination than mice treated with two injections of anti-TNFR1 (**E**, image from animal with an EAE score of 0.5), although this was not found to be statistically significant. Immunohistochemistry with an anti-CD3 antibody showed no difference in the number of T cells within the spinal cord of control IgG-treated animals (**F**, **G**, image from an animal with an EAE score of 2.5) and anti-TNFR1 treated mice (**H**, image from an animal with an EAE score of 2.0). Immunohistochemistry with an antibody to Mac-3 also demonstrated no difference in the number of activated microglia/macrophages within the spinal cord of control IgG-treated animals (**I**, **J**, image from an animal with an EAE score of 2.5) and anti-TNFR1 treated mice (**K**, image from an animal with an EAE score of 2.0). **L**–**N**: Immunohistochemistry with an antibody against NeuN showed a statistically non-significant trend towards neuroprotection in the anti-TNFR1-treated mice (**M**, image from a control IgG-treated animal with an EAE score of 1.5), (**N**, image from an anti-TNFR1-treated animal with an EAE score of 1.5). Scale bars in **D**, **E, G, H, J, K, M, N**: 200 µm. * P<0.05. Control IgG n = 9; anti-TNFR1: 1 injection n = 4; anti-TNFR1: 2 injections n = 8.

Whereas no effect was observed with dosages of 100, 200 or 300 µg (data not shown), a single treatment with 400 µg at the day of disease onset ameliorated the disease for several days (Fig. 6A). Using this dosage we then evaluated whether an additional dose of anti-TNFR1 could prolong the therapeutic effect. Indeed, application of a second dose of 400 µg at day 4 of EAE resulted in a stable, even slightly reduced, disease score for at least seven additional days and thus led to a significantly reduced cumulative EAE score (Fig. 6A, B). Histopathological analysis of spinal cords revealed a reduction in the level of spinal cord demyelination, although, with the applied short treatment protocol, the observed differences did not reach statistical significance at day 14 after disease onset (Fig. 6C–E). Further, at this time point (10 days after the last antibody injection), no influence on T cell infiltration (Fig. 6F–H) and number of activated macrophages/microglia (Fig. 6I–K) was observable, corresponding to a beginning rise in EAE score again. Similarly, a trend towards a reduction in neuronal loss, as demonstrated by NeuN staining of surviving grey matter neurons of the spinal cord, was seen but without significance (Fig. 6L–N). Thus, therapeutic administration of anti-TNFR1 appears to be more effective than prophylactic administration in reducing disease severity (in the short term at least). This might be explained by neurodegenerative processes which may have already begun during the induction phase of the disease [34]. However, despite both protocols resulting in moving the development of EAE towards a reduction in both demyelination and neurodegeneration, this was less apparent in the therapeutic setting, demonstrating that improvements in the methodology of antibody administration are still needed to have the best impact in a clinical setting.

## Discussion

In this study we have used a neutralizing TNFR1-specific antibody to assess the potential therapeutic role of TNFR1 inhibition in an inflammatory neurodegenerative disease using an animal model of MS. Using MOG^35–55^-induced EAE, we show that a single injection of anti-TNFR1 at the time of immunization is sufficient to significantly delay and ameliorate EAE symptoms and promote neuroprotection in C57Bl/6 mice. Moreover, in a therapeutic setting, two sequential injections of anti-TNFR1 early after disease onset reduced EAE symptoms to a very moderate score (0,5) for about two weeks.

In preliminary experiments, we showed that TNFR1^-/-^ C57BL/6 mice are almost completely protected from EAE. The lack of EAE symptoms was reflected in very low levels of activated microglia/macrophages and infiltrating T cells in the CNS parenchyma and accordingly low levels of demyelination and neurodegeneration. In contrast, TNFR2^-/-^ mice showed a significantly increased EAE disease course compared to WT mice together with significantly higher levels of neurodegeneration. In general, our results with TNFR deficient mice confirm previous findings that TNFR1 is required for EAE development whereas TNFR2 has a neuroprotective role [30]–[32]. In fact, a protective role for TNFR2 has been determined both *in vitro* where TNFR2 protected neurons from excitotoxic insults [35], [36] and *in vivo* where neuronal and oligodendrocyte survival was promoted following ischemic and neurotoxic insults, respectively [19], [20]. This is in agreement with our finding that TNFR2^-/-^ mice had greater axonal damage in EAE. It remains a possibility that pan-TNF targeting strategies, which have resulted in the promotion or exacerbation of disease in MS patients, have failed due to their inability to leave protective TNFR2 functioning whilst targeting only the pro-inflammatory TNFR1 pathway. Thus, the most promising approach for clinical application in inflammatory demyelinating diseases may be the direct targeting of TNFR1 with a specific antagonist [37], [38]. However, based on past experiences any extrapolation to the human condition must be made with care.

In support of the role of TNFR1 in EAE development, R1antTNF, a TNFR1 specific antagonistic TNF mutant, ameliorates MOG^35–55^-induced EAE in a prophylactic setting [23]. More recent data suggest that R1antTNF acts via formation of heterotrimers with endogenous TNF [39], thus closely resembling independently developed soluble dominant-negative TNF variants [40], one of which, XPro1595, was also recently shown to exert beneficial effects in EAE [23], [24]. The formation of inactive heteromeric TNF molecules was shown for processed, soluble TNF and supports the notion that TNF signaling via TNFR1 mediates neuroinflammation in EAE, since soluble TNF predominantly activates TNFR1 [16]. However, since both R1antTNF and XPro1595 primarily target soluble TNF but do not interfere with the signaling of the transmembrane form, which can also activate TNFR1, we reason that a direct inhibition of TNFR1 should be a preferential therapeutic approach in neuroinflammatory diseases.

Furthermore, a single nucleotide polymorphism (SNP) identified in the largest MS genome-wide association study [41], has recently been reported as occurring in the *TNFRSF1A* gene, that encodes TNFR1, and has been shown to be associated with an increased susceptibility to MS development. It is now understood that this SNP results in the expression of a soluble form of TNFR1, which can block pan TNF signalling [42]. Treatment using an anti-TNFR1 a strategy might also neutralize the detrimental effects of this soluble TNFR1 variant.

Moreover, considering a potential clinical application, one shortcoming of TNF based antagonists is their relatively short *in vivo* half-life, which typically is in the range of less than 1 hour. In contrast, the TNFR1 selective antibody antagonist used here has a half-life of approximately 2 days [28], which is clearly superior to cytokines. To block TNFR1 signaling, we have therefore used this mouse-TNFR1-specific antibody, and confirmed its TNFR1 selective antagonistic activity *in vitro* as well as in an *in vivo* TNF-mediated shock model.

Using this antibody in a prophylactic setting in EAE, a single injection of 100 µg (5 mg/kg) at the time of immunization was sufficient to significantly delay and ameliorate the EAE disease course. Given the 2 day half-life of this antibody [29], the observed disease modulation in this setting is predominantly due to the inhibition of TNF-TNFR1 signalling in the induction phase of the disease. This may result in a reduced activation of macrophages and expansion of CNS-directed T cells in the peripheral immune system, e.g. by impaired activation of dendritic cells [26], [27]. The inhibition of TNFR1 signalling during the induction phase of EAE may also result in a reduced infiltration of T cells into CNS tissue. TNFR1 signalling is also known to be required for the infiltration of both Th1 and Th17 cells into the spinal cord [22] possibly via the regulation of VCAM-1 expression on astrocytes [43]. These mechanisms would also reflect the slightly reduced amount of these cells in the chronic stage of EAE. Importantly, the reduced EAE score was accompanied with a significantly reduced demyelination and neurodegeneration in the chronic stage of the disease thus demonstrating the effectiveness of anti-TNFR1 treatment to reduce tissue degeneration in EAE.

The effects of the TNFR1-specific antibody on the EAE course in this prophylactic setting are to some extent comparable to those of the inhibitors of soluble TNF, R1antTNF and XPro1595. R1antTNF treatment ameliorated EAE clinical score, which was accompanied by reduced demyelination and T cell infiltration at the peak of disease. However, in accordance with its inferior pharmacokinetic properties, R1antTNF had to be injected twice daily to achieve this effect [22]. Similar to anti-TNFR1 treatment, injection of the dominant-negative TNF XPro1595 twice weekly caused a delay of EAE development of approximately four days. However, this treatment was only partially sufficient in ameliorating the EAE clinical score in this prophylactic setting [24].

Importantly, anti-TNFR1 treatment could significantly reduce EAE symptoms when applied in a therapeutic setting, i.e. on the day of disease onset. However, a four-fold higher antibody concentration compared to the prophylactic setting and two subsequent injections (day 1 and 4) were required to achieve a longer-lasting therapeutic effect, which could be discerned for a two week period. Different modes of action of the TNFR1 antagonist in the two treatment regimens may account for the observed differences. Whereas in the prophylactic setting the antibody has direct access to the immune system and can thus directly inhibit TNF-TNFR1 signalling, in the therapeutic setting T cells and macrophages have already penetrated the blood-brain barrier (BBB) and demyelination and neurodegeneration have started. Therefore, to achieve a therapeutic effect in this setting, the antibody has to penetrate the BBB. Although the BBB is compromised in EAE, it is likely that only a limited amount of the antibody will get access to the perivascular space and/or the CNS parenchyma explaining the high dosage required for the therapeutic effect. Moreover, the long term availability of this hamster antibody, determined by its basic half-life of 2 days and its potential later elimination due to antidrug antibody responses of the host to this xenogenic protein are apparent limitations in achieving long term remissions in this model system.

In general, our results are similar to the effect of XPro1595, which also ameliorated the EAE clinical score in a therapeutic setting [24], although there the effect was less pronounced compared to the treatment with anti-TNFR1. At first sight it may be surprising that we did not find a significant reduction of the amount of infiltrating lymphocytes and activated macrophages in sections of the spinal cord after treatment with anti-TNFR1. However, this result is consistent with data from studies, both with XPro1595 [24] and a TNFR1 fusion protein [44], [45] indicating that the therapeutic effect of anti-TNFR1 treatment after disease onset is not due a modulation of the peripheral immune system, but instead might be due to neuroprotective activity [23], [24]. Although, in principle, transmembrane TNF can activate both TNFR1 and TNFR2, our finding that anti-TNFR1 has similar effects to XPro1595, therefore may support the notion that blocking of TNFR1 is neuroprotective in EAE. An intriguing second possibility is that neutralizing TNFR1 may indirectly promote stimulation of TNFR2 by TNF present in the EAE lesions. There is ample evidence that TNF-signalling via TNFR2 is *in vitro* and *in vivo* neuroprotective and can promote remyelination in chemically-induced demyelination [19], [20], [35], [36], [46], [47]. Moreover, TNF-signalling via TNFR2 can promote the release of neurotrophic factors from microglia [48].

In conclusion, our finding that the selective neutralization of TNFR1 significantly ameliorates EAE in both prophylactic and therapeutic settings provides further experimental evidence to support the hypothesis that TNFR1 is responsible for the detrimental role of TNF in neuroinflammatory disorders. Our results also further the idea that applications of TNFR1-selective antibodies have a significant therapeutic potential in the treatment of these diseases.
